# Global analysis of tRNA and translation factor expression reveals a dynamic landscape of translational regulation in human cancers

**DOI:** 10.1038/s42003-018-0239-8

**Published:** 2018-12-21

**Authors:** Zhao Zhang, Youqiong Ye, Jing Gong, Hang Ruan, Chun-Jie Liu, Yu Xiang, Chunyan Cai, An-Yuan Guo, Jiqiang Ling, Lixia Diao, John N. Weinstein, Leng Han

**Affiliations:** 1 Department of Biochemistry and Molecular Biology, McGovern Medical School at The University of Texas Health Science Center at Houston, Houston, TX 77030 USA; 2 Department of Bioinformatics and Systems Biology, Hubei Bioinformatics and Molecular Imaging Key Laboratory, Key Laboratory of Molecular Biophysics of the Ministry of Education, College of Life Science and Technology, Huazhong University of Science and Technology Wuhan, 430074 Hubei, People’s Republic of China; 3 Department of Internal Medicine, McGovern Medical School at The University of Texas Health Science Center at Houston, Houston, TX 77030 USA; 4 Department of Cell Biology and Molecular Genetics, University of Maryland, College Park, MD 20742 USA; 5 Department of Bioinformatics and Computational Biology, The University of Texas MD Anderson Cancer Center, Houston, TX 77030 USA; 6 Center for Precision Health, The University of Texas Health Science Center at Houston, Houston, TX 77030 USA

**Keywords:** Cancer, Computational biology and bioinformatics, Cancer genomics

## Abstract

The protein translational system, including transfer RNAs (tRNAs) and several categories of enzymes, plays a key role in regulating cell proliferation. Translation dysregulation also contributes to cancer development, though relatively little is known about the changes that occur to the translational system in cancer. Here, we present global analyses of tRNAs and three categories of enzymes involved in translational regulation in ~10,000 cancer patients across 31 cancer types from The Cancer Genome Atlas. By analyzing the expression levels of tRNAs at the gene, codon, and amino acid levels, we identified unequal alterations in tRNA expression, likely due to the uneven distribution of tRNAs decoding different codons. We find that overexpression of tRNAs recognizing codons with a low observed-over-expected ratio may overcome the translational bottleneck in tumorigenesis. We further observed overall overexpression and amplification of tRNA modification enzymes, aminoacyl-tRNA synthetases, and translation factors, which may play synergistic roles with overexpression of tRNAs to activate the translational systems across multiple cancer types.

## Abstract (web summary)

Zhao Zhang, Youqiong Ye et al. present an expression analysis of tRNAs and genes encoding enzymes involved in translation regulation across ~10,000 patients from 31 cancer types in The Cancer Genome Atlas (TCGA). They find that cancer cells may overcome translational bottlenecks by overexpressing rarer tRNAs and other translation factors.

## Introduction

Translational regulation is critical for biological functions and cellular processes^1–4^. In the translational system, transfer RNAs (tRNAs) play essential roles by delivering amino acids to initiate or elongate a peptide chain on the ribosome^5^, and they account for ~10% of total cellular RNAs by weight^6^. The human genome includes approximately 600 annotated tRNA genes, which code for 62 codons and 21 amino acids^7,8^. Activation of the oncogenic signaling pathways^9^, including AKT-mTOR, RAS-MAPK, and MYC or loss of the tumor suppressor *TP53* can regulate RNA polymerase III expression^10–13^, thus leading to altered tRNA expression. In general, overexpression of tRNAs may enhance tumor progression by supplying the high demand codons of oncogenic pathways^14,15^. Despite the essential functions of tRNAs in the cell, it is still challenging to perform high-throughput quantification of tRNAs, mainly due to the presence of post-transcriptional modifications and secondary structures^16^. To address these challenges, several methods have been designed to quantify tRNA expression level, including tRNA microarrays, which can only achieve codon level resolution by recognizing the tRNA’s anticodon loop^17–19^, and tRNA-sequencing methods, such as demethylase-tRNA-seq (DM-tRNA-seq)^16^, which has been applied in a few cell lines^16,20,21^. Alternatively, it is also possible to quantify tRNA expression from miRNA-sequencing (miRNA-seq), which has been applied in small patient sample cohorts^22–29^. These methods have previously not been applied in large numbers of cancer patient samples.

Multiple categories of enzymes are involved in translational regulation, including the tRNA modification enzymes, aminoacyl tRNA synthetases (ARSs), and translation factors. The first category, tRNA modification enzymes, maintains the stability and specificity of tRNA structure by chemically modifying tRNAs post-transcriptionally^30,31^. Several modification enzymes, including those encoded by *NSUN2*, *TRMT12*, and *TRMT2A* have been reported to serve as oncogenes^32–35^, and others, including *RG9MTD2*, *KIAA1456*, and *TRDMT1*, serve as tumor suppressors^36–38^. However, there is still a lack of knowledge about the majority of tRNA modification enzymes in cancer. The second category, the ARSs, includes cytosolic ARSs (cy-ARSs) and mitochondrial ARSs (mt-ARSs). ARSs function to attach the appropriate amino acids to their respective unloaded tRNAs, thereby initiating or elongating the peptide chain by recognizing the codon in the messenger RNA sequence. Cy-ARSs are involved in tumorigenesis through their interaction with aminoacyl tRNA synthetase-interacting multifunctional proteins to influence cancer cell proliferation and oncogenic transformation^39^. Alterations in the expression of multiple cy-ARSs, such as *CARS*, *IARS*, and *YARS*, are involved in tumorigenesis by promoting oncogenic pathways^40–42^, but it is unclear whether the mt-ARSs are altered and involved in tumorigenesis. The third category, translation factors, that mediate translational initiation and translational elongation, also play important roles in cancer^43^. For example, *EIF3H* is up-regulated in prostate cancer^44^, *EIF4G* is up-regulated in lung cancer^45^, whereas *EIF3F* is down-regulated in melanoma and pancreatic cancer^46,47^. Previous studies have generally described only one category of enzymes or even individual enzymes based on relatively small sample cohorts.

The Cancer Genome Atlas (TCGA) provides a uniquely comprehensive data resource, including ~10,000 humans patients^48^. In this study, we performed a comprehensive analysis elucidating a dynamic landscape of translational regulation, including tRNAs, tRNA modification enzymes, ARSs, and translation factors, across multiple cancer types in TCGA. Our results highlight a synergistic activation of the translational system in cancer.

## Results

### Expression landscape of tRNAs across 31 cancer types

We obtained tRNA annotations from the UCSC genome browser (http://genome.ucsc.edu/), including 604 tRNA transcripts, 62 codons, and 21 amino acids. We then mapped those reads obtained from miRNA-seq to tRNA annotation to infer the relative expression level of tRNAs. The tRNA expression data were merged to the codon level and amino acid level according to the anticodon and amino acid information (Supplementary Figure 1A). We first analyzed DM-tRNA-seq^20^ and miRNA-seq^49^ data from 293T cells to test our computational pipeline. Our analysis showed a high correlation at the tRNA level (Spearman's correlation *R*s *=* 0.73, *p* < 2 × 10^−16^), codon level (*R*s *=* 0.61, *p* = 7.2 × 10^−7^), and amino acid level (*R*s *=* 0.59, *p* = 4.1 × 10^−3^, Supplementary Figure 1B) between both data types, indicating the reliability of our computational pipeline to infer the relative expression levels of tRNAs.

**Fig. 1:**
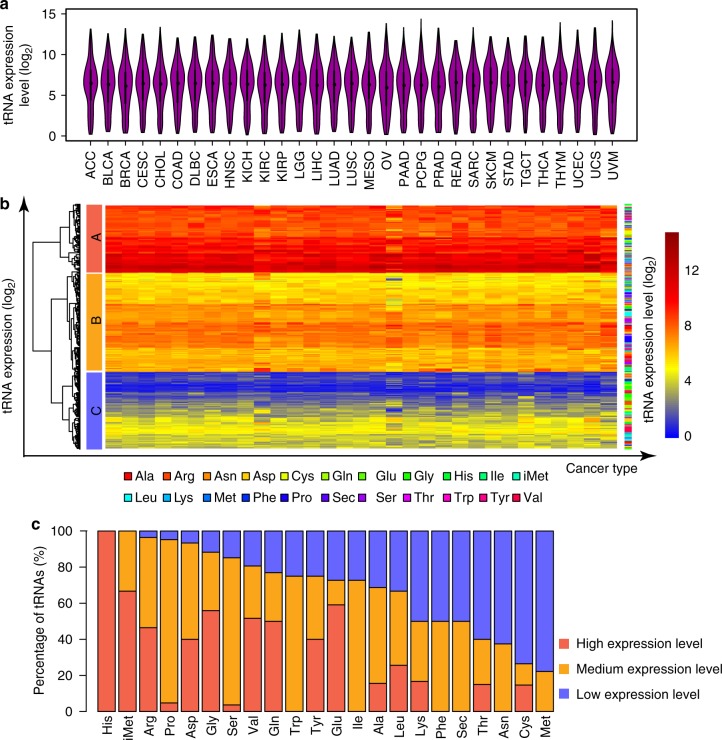
Overview of tRNA expression across multiple cancer types. **a** Distribution of tRNA expression value (*Y*-axis, log_2_ TMM) across 31 cancer types (*X*-axis). Please refer Supplementary Figure 1D and Supplementary Table 1 for full cancer names. For each violin plot, the center dot represents median; the bold line represents 1.5× interquartile range. **b** Clustering of tRNA expression across cancer types. Nodes A, B, and C, respectively, denote the high-, middle-, and low-expression groups. *X*-axis represents 31 cancer types and *Y*-axis represents the expression value of tRNA genes (log_2_(TMM)). The right colored bar stands for 22 tRNA^amino acids^. **c** Percentage of tRNAs in different groups based on their expression group in **b**. Different tRNA genes for the same amino acid or function (i.e., Met divide into iMet and Met base on their divergent function) are grouped together

To comprehensively analyze the expression profiles of tRNAs from TCGA^48^, we downloaded all miRNA-seq samples across 31 cancer types from the TCGA data portal (https://portal.gdc.cancer.gov/). After filtering out duplicated and low-quality samples, we retained 9931 cancer samples and 663 normal samples for analysis (Supplementary Figure 1C). The number of samples and detailed abbreviations for each cancer type are listed in Supplementary Figure 1D and Supplementary Table 1. We detected the expression of 490 distinct tRNA genes (trimmed mean of *M* values, TMM > 1) among the multiple cancer types. This figure accounts for 81.1% (490/604) of all annotated tRNA genes in the human genome. The average number of reads per detectable tRNA ranged from 2 to 9558, with the median as 141. The log_2_ expression values (log_2_ TMMs) of tRNA genes ranged from 0.13 to 14.36 with a median of 6.11 (5.86–6.40 for the different cancer types; Fig. 1a). The different cancer types showed strikingly similar overall average expression levels and patterns of tRNA expression (Fig. 1a). We were able to classify tRNAs into three groups by unsupervised clustering (Fig. 1b): 135 high-expression genes (cluster A), with a median expression of tRNA genes across cancer types ≥ 7.88; 200 medium-expression genes (cluster B), with median expression values between 4.99 and 7.88; and 155 low-expression genes (cluster C), with a median expression ≤ 4.99.

The primary function of tRNAs is to carry amino acids to the ribosome to initiate and elongate growing peptides^50^. We analyzed tRNAs based on the amino acids they accepted. The number of tRNA genes detected for each amino acid ranged from two for selenocysteine (Sec) to 39 for leucine (Leu) (Supplementary Figure 2). The tRNA expression levels for each amino acid varied greatly. For example, all tRNA genes for histidine (His) were highly expressed, and more than 90% of tRNA genes for arginine (Arg), proline (Pro), aspartic acid (Asp), and the methionyl initiator of translation (iMet) were high or moderate in expression. In contrast, more than 60% of tRNA genes for cysteine (Cys), asparagine (Asn), threonine (Thr), and methionine (Met) were low in expression. In particular, none of the tRNA genes for tryptophan (Trp), leucine (Leu), phenylalanine (Phe), Asn, or Sec were in the high-expression cluster (Fig. 1c). Taken together, our results reveal a diverse transcriptional landscape for different tRNAs at the tRNA level across multiple cancer types in more than 10,000 samples.

### Alterations of tRNA gene expression across cancer types

**Fig. 2:**
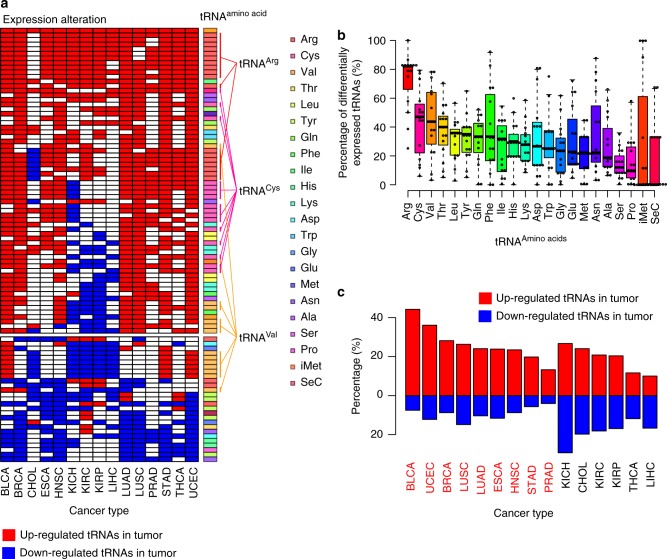
Differential expression of tRNA genes across different cancer types. **a** tRNAs differentially expressed in at least eight cancer types. Red denotes up-regulation; blue denotes down-regulation (|fold-change| ≥ 1.5, FDR < 0.05). *X*-axis represents 15 cancer types with > 5 tumor-normal paired miRNA-seq samples. The color bar denotes to tRNAs based on their decoded amino acids or function. **b** Percentage of differentially expressed tRNAs among amino acid groups. Each dot denotes a cancer type. The boxes show the median ± 1 quartile, with whiskers extending to the most extreme data point within 1.5 interquartile range from the box boundaries. **c** Percentage of up-regulated (red bars) and down-regulated (blue bars) tRNAs across different cancer types. *X*-axis represents 15 cancer types with > 5 tumor-normal paired miRNA-seq samples

To systematically understand the potential functions of tRNAs in tumorigenesis, we examined the differences in tRNA gene expression levels between paired tumor and normal samples. We identified a total of 474 differentially expressed tRNA genes (96.7% of the 490 detectable tRNA genes) across the 31 cancer types (Supplementary Data 1). There were 93 tRNAs with pervasive differential expression in at least eight cancer types (Fig. 2a). Among them, 66 tRNA genes were pervasively up-regulated. The spectrum showed 18 tRNA^Arg^, 16 tRNA^Cys^, and 7 tRNA^Val^ with pervasive overexpression, as well as 25 tRNAs that carry 11 other amino acids. At the other end of the spectrum, 27 pervasively down-regulated tRNAs including six tRNA^Val^ carrying nine amino acids. Of interest, tRNA^Val^ showed up-regulation in nine cancer types and down-regulation in five cancer types, suggesting cancer-specific features of tRNA^Val^ ^51–53^. Interestingly, tRNA^Arg^, tRNA^Cys^, and tRNA^Val^ showed the highest percentage of altered expression across multiple cancer types (Fig. 2b), suggesting their functional roles in tumorigenesis. We observed 1518 (20.7%) up-regulated and 880 (12.0%) down-regulated tRNAs (|fold-change| ≥ 1.5, false discovery rate (FDR) < 0.05) across different cancer types (Supplementary Data 1). Nine cancer types, including bladder cancer (BLCA), uterine corpus endometrial carcinoma (UCEC), and breast cancer (BRCA), showed predominant up-regulation of tRNA expression. Six cancer types, including kidney chromophobe (KICH), cholangiocarcinoma (CHOL), and kidney renal clear cell carcinoma (KIRC), showed similar numbers of up-regulated and down-regulated tRNA genes (Fig. 2c). That observation suggests the overall overexpression of tRNAs at the tRNA level across multiple cancer types.

### Alterations of tRNA gene expression at the codon level

**Fig. 3:**
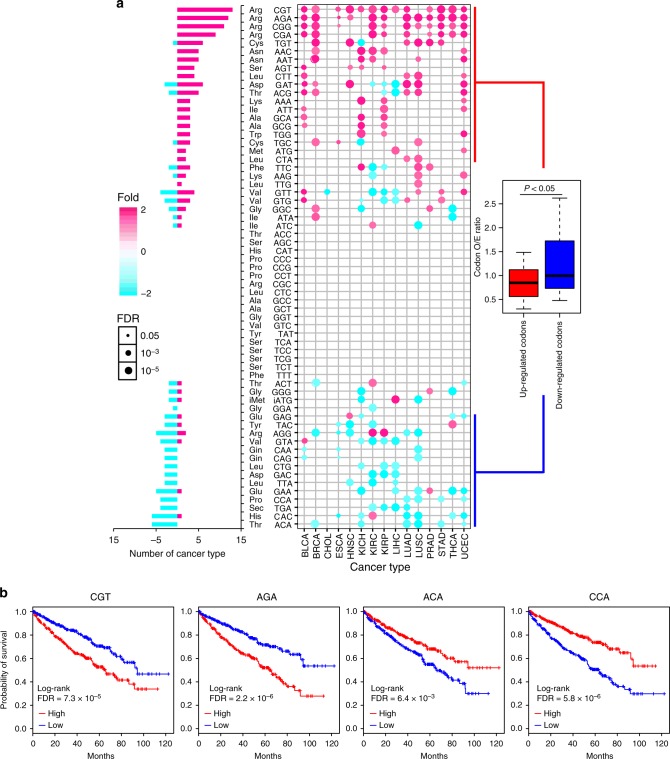
Differential expression of tRNAs at codon level and functional consequences. **a** Differentially expressed tRNAs at codon level across different cancer types. Magenta denotes up-regulation; cyan denotes down-regulation. Color bar in the left panel denotes the number of cancer types with differentially expressed codons. X-axis represents 15 cancer types with > 5 tumor-normal paired miRNA-seq samples and *y*-axis is tRNA at the codon level (middle panel). Circles denote tRNAs with expression alterations. The boxplot (right panel) shows the comparison of codon O/E ratio between up-regulated codons and down-regulated codons (Student’s *t* test, *p* = 0.047; Wilcoxon's test, *p* = 0.043; bootstrap test, *p* = 0.017). **b** Survival curves for KIRC patients with two up-regulated codons and two down-regulated codons. Red and blue curves denote patients with high and low tRNA expression, respectively. The log-rank test was used to examine the association between tRNA expression at the codon level and patient survival

tRNAs deliver amino acids to initiate and elongate peptide chains by recognizing specific codons^54^. To further explore the possible functions of tRNAs in cancer, we merged tRNA genes to the codon level. Forty-five (72.5%) codons showed significantly differential expression (|fold-change| ≥ 1.5, FDR < 0.05) in at least one cancer type (Fig. 3a and Supplementary Data 2). Eight cancer types (including BLCA, UCEC, and BRCA) showed predominantly up-regulated codons, whereas two cancer types (CHOL and liver hepatocellular carcinoma) showed predominantly down-regulated codons. Five cancer types (including LUSC, KICH, and KIRC) showed similar numbers of up- and down-regulated codons (Supplementary Figure 3A).

tRNAs for nine codons (represented here as tRNA^amino acid^(codon)) were pervasively up-regulated in at least five cancer types. Included were four Arg codons (tRNA^Arg^(CGT), tRNA^Arg^(AGA), tRNA^Arg^(CGG), and tRNA^Arg^(CGA)). Similarly, four tRNAs codons (tRNA^Thr^(ACA), tRNA^His^(CAC), tRNA^Glu^(GAA), and tRNA^Arg^(AGA)) were pervasively down-regulated (Fig. 3a). To understand the effects of codon usage frequency on their expression, we used the observed-over-expected ratio (O/E ratio) to represent the codon usage frequency. Interestingly, those codons that tend to be overexpressed in cancer samples showed significantly lower O/E ratio than those codons that tend to be down-regulated (Fig. 3a, Student’s *t* test *p* = 0.047; Wilcoxon's test *p* = 0.043; bootstrap test *p* = 0.017), suggesting that overexpression of codons with low O/E ratio may overcome the bottleneck in tumor development.

We next asked whether tRNA expression at the codon level has prognostic value, and found that the expression of several codons was correlated with patient survival times across different cancer types (Supplementary Figure 3A, Cox's model). For example, overexpression of multiple codons, including tRNA^Arg^(CGT) (two-sided log-rank test, FDR = 7.3 × 10^−5^) and tRNA^Arg^(AGA) (FDR = 2.2 × 10^−6^), were associated with worse survival in KIRC (Fig. 3b). In contrast, down-regulation of several codons, including tRNA^Thr^(ACA) (FDR = 6.4 × 10^−3^) and tRNA^Pro^(CCA) (FDR = 5.8 × 10^−6^), were associated with worse survival (Fig. 3b). These results suggest the possibility of tRNA expression levels to serve as a prognostic marker.

### Unequal alterations at tRNA codon and amino acid levels

**Fig. 4:**
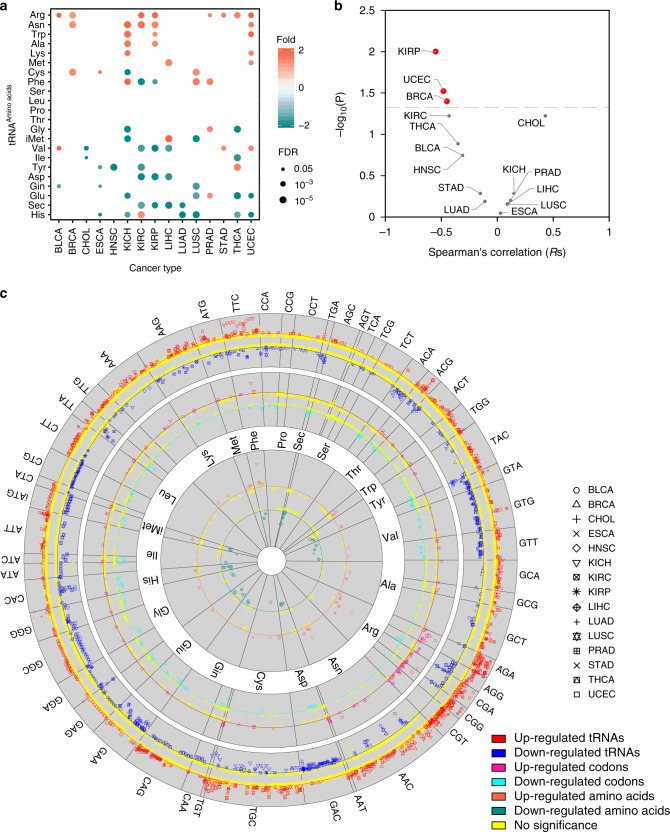
Differential expression of tRNAs at the amino acid level and functional consequences. **a** tRNAs differentially expressed at the amino acid level across different cancer types. Tomato denotes up-regulation; dark cyan denotes down-regulation. *X*-axis represents 15 cancer types with > 5 tumor-normal paired miRNA-seq samples. Circles denote tRNAs with expression alterations. **b** Scatter plot for Spearman's correlation between amino acid O/E ratio and tRNA expression alterations at the amino acid level across 15 cancer types. Significant correlations are highlighted in red. **c** Unequal expression alterations of detectable tRNA at gene, codon, and amino acid levels. Outer, middle, and inner circles denote the tRNA level, codon level, and amino acid level, respectively. The angle subtended by each cell is scaled by the number of tRNA genes. Different shapes denote different cancer types. Red, magenta, and tomato denote significant up-regulation (fold-change  ≥ 1.5, FDR < 0.05) at the tRNA level, codon level, and amino acid level, respectively; blue, cyan, and dark cyan denote significant down-regulation at tRNA, codon, and amino acid levels, respectively. Yellow denotes no significant difference. Cancer types were denoted in different shapes. The figure was drawn by R package circlize

We examined alterations of tRNA gene expression at the amino acid level and observed diverse patterns across different cancer types. tRNA^Arg^ was pervasively up-regulated in eight cancer types, and tRNA^Asn^ was up-regulated in five cancer types (Fig. 4a and Supplementary Data 3). Those findings are consistent with their up-regulation at the tRNA and codon levels. In contrast, tRNA^His^ was down-regulated in at least five cancer types. tRNA^Ser^, tRNA^Thr^, tRNA^Pro^, and tRNA^Leu^ showed no significant alterations of expression (Fig. 4a). Interestingly, tRNA^Val^ showed significant up-regulation in three cancer types and down-regulation in four cancer types, further confirming that tRNA^Val^ may have divergent functions across different cancer types.

We analyzed the correlations between alterations at the amino acid expression level and the observed-over-expected ratio (O/E ratio) across cancer types. Interestingly, we observed a significantly negative correlation between expression alterations and the amino acid O/E ratio in multiple cancer types (Fig. 4b), such as in kidney renal papillary cell carcinoma (KIRP) (*R*s = −0.55, *p* = 0.01, Supplementary Figure 4). Other studies have demonstrated that tRNA^Arg^ is up-regulated in cancer cells^17,18^, and that overexpression of tRNA^Arg^ enhances the ability of cancer cells to invade other tissues and metastasize^17^. It apparently does so by increasing the codon-dependent stability and translation of genes with high Arg codon content^17^. For example, tRNA^Arg^ was up-regulated in breast cancer, and the oncogene *TERT* protein was also up-regulated in breast cancer samples^55^, while *TERT* has significantly higher Arg usage frequency (O/E = 1.98) compared to the genomic Arg usage frequency (O/E = 0.57). These results suggest that overexpression of a rare amino acid could facilitate the overexpression of those genes with high amino acid usage frequency, thus overcoming the bottleneck in tumor development.

In general, alterations at the tRNA level (defined as tRNA^amino acid^_anticodon_) will lead to alterations at the codon level (defined as tRNA^amino acid^ (codon)) and at the amino acid level (defined as tRNA^amino acid^). For example, our results showed consistent up-regulation of tRNA^Arg^ at the tRNA level, codon level, and amino acid level, suggesting that they may function as oncogenes (Fig. 4c). We observed consistent down-regulation for tRNA^His^ and tRNA^Glu^, suggesting that they might act as tumor suppressor genes. Despite connections among tRNAs, codons, and amino acids, the tRNA expression alterations at the three levels also appeared inconsistently with each other. That inconsistency is probably due to the uneven distribution of tRNAs and codons. Each amino acid has one (e.g., Trp, Met) to five (e.g., Arg, Leu, Ser) detectable codons (Supplementary Figure 5A), and each codon has 1 (e.g., tRNA^Cys^(ACA)) to 31 (e.g., tRNA^Cys^(GCA)) detectable tRNA genes (Supplementary Figure 5B). We observed expression alteration at the tRNA level but not in the codon or amino acid level for two possible reasons. First, alteration of tRNAs in opposite directions may lead to unaltered expression at the codon level or amino acid level. For example, tRNA-Tyr-GTA-5-3 and tRNA-Tyr-GTA-9-1 were up-regulated, whereas tRNA-Tyr-GTA-6-1 was down-regulated in BRCA, leading to no significant difference for tRNA^Tyr^(TAC) and tRNA^Tyr^ (Fig. 4c and Supplementary Data 1–3). Second, the alteration of only a few tRNAs may not be sufficient to imply significant alterations at the codon or amino acid level. For example, in KIRC, we observed 3/16 (18.8%) up-regulated tRNA^Ala^_AGC_ but no alterations in the expression of tRNA^Ala^(GCT) or tRNA^Ala^ (Fig. 4c and Supplementary Data 1–3). Through this comprehensive analysis, we revealed unequal alterations at multiple levels, which is largely due to the uneven distribution of tRNAs and codons.

### Dynamic landscape of enzymes involved in translation

**Fig. 5:**
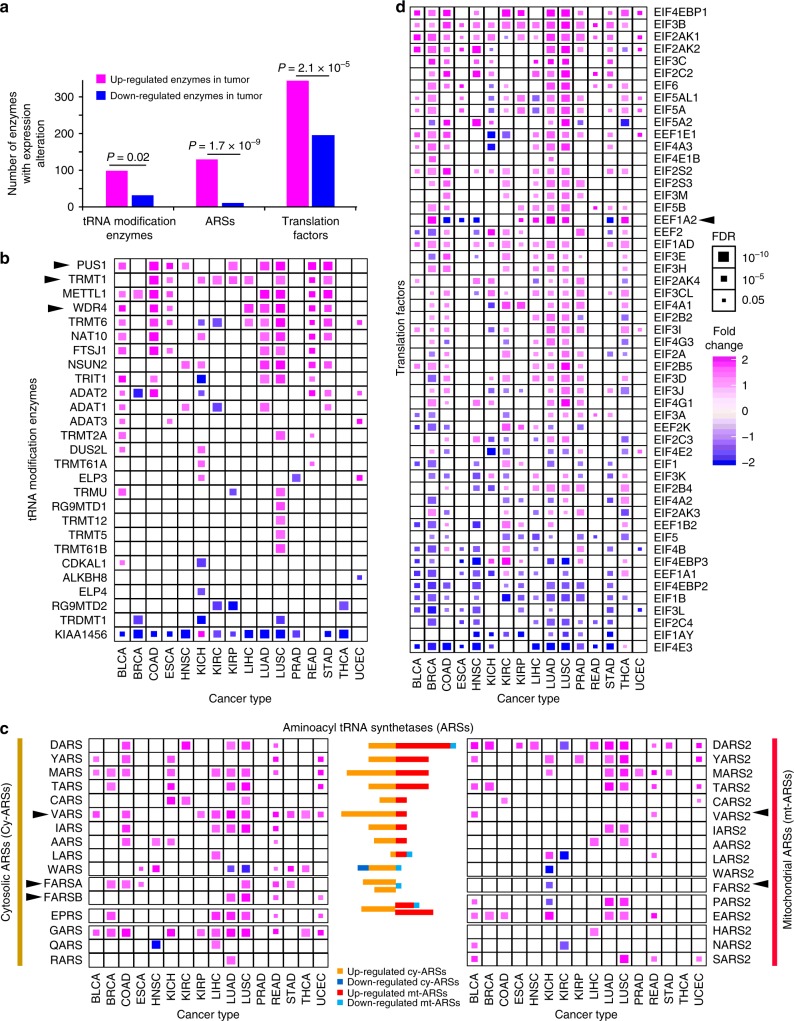
Expression alteration of tRNA modification enzymes, ARSs, and translation factors across cancer types. **a** Two-sided *χ*^2^ test for up-regulated and down-regulated enzymes for tRNA modification enzymes (left), aminoacyl tRNA synthetases (middle), and translation factors (right). **b** Differentially expressed tRNA modification enzymes between paired tumor and normal samples. Magenta denotes up-regulation; blue denotes down-regulation. *X*-axis represents 16 cancer types with > 5 tumor-normal paired RNA-seq samples. Squares denote enzymes with expression alterations. **c** Differentially expressed aminoacyl tRNA synthetases (ARSs) between paired tumor and normal samples. Left panel, cytosolic ARSs (cy-ARSs); right panel, mitochondrial (mt-ARSs). The color bars in the middle panel summarize the comparisons between paralog ARSs. Squares denote enzymes with expression alterations. **d** Differentially expressed translation factors between paired tumor and normal samples. Squares denote enzymes with expression alterations

Numerous categories of enzymes, including tRNA modification enzymes, ARSs, and translation factors, are involved in translational regulation. To gain mechanistic insights into how these enzymes are altered in cancer, we examined the gene expression landscape and copy number variations (CNVs) of these enzymes. For tRNA modification enzymes, we observed a total of 97 up-regulated enzymes compared to a total of 30 down-regulated enzymes, suggesting the overall up-regulation of these enzymes across different cancer types (two-sided *χ*^2^ test, *p* = 0.02, Fig. 5a, left panel; Supplementary Data 4). We found that 20/29 (69.0%) tRNA modification enzymes showed up-regulation in at least one cancer type (Fig. 5b). For example, *METTL1* has been reported to promote lung cancer^56^, while we observed significant overexpression of *METTL1* in nine cancer types, suggesting it as the master oncogenic event. In addition, we identified several novel oncogenic enzymes with up-regulation in multiple cancer types, including *PUS1*, a tRNA pseudoridylate synthase, and *TRMT1* and *TRMT6*, the tRNA methyltransferases. These enzymes play essential roles to maintain tRNA structure through modifying certain nucleotide residues. In contrast, several enzymes, including *KIAA1456*, *RG9MTD2*, and *TRDMT1*, showed down-regulation and can potentially act as tumor suppressors^36–38^. Consistent with the overall overexpression pattern, we also observed overall amplification of CNV for tRNA modification enzymes, including those encoded by *TRMT12*, *NSUN2*, and *TRMT6* (Supplementary Figure 6A). Taken together, the overall overexpression of tRNA modification enzymes may stabilize tRNAs to facilitate overexpression of tRNAs.

In analyzing 37 ARSs, including 20 cy-ARSs and 17 mt-ARSs, across different cancer types, we observed a total of 128 up-regulated and 9 down-regulated enzymes (two-sided *χ*^2^ test, *p* = 1.7 × 10^–9^, Fig. 5a, middle panel). Specifically, we observed overall up-regulation across different cancer types, with 31 out of 37 ARSs (83.8%), exemplified by *GARS* and *VARS*, up-regulated in at least one cancer type (Fig. 5c). Among these, mt-ARSs, which have been neglected by previous studies, also showed abundant up-regulation across different cancer types. Consistent with the overall overexpression pattern, we also observed overall copy number amplifications for ARSs, including those encoded by *TARS2*, *TARS*, and *DARS2* (Supplementary Figure 6B). In particularly, *GARS* consistently showed overexpression and copy number amplification in colon adenocarcinoma (COAD), KICH, KIRP, lung adenocarcinoma, and rectum adenocarcinoma (READ). Interestingly, the isoenzymes in the cytoplasm and mitochondria may show very distinct expression patterns. For example, *VARS* is up-regulated in 10 cancer types, whereas *VARS2* is only up-regulated in two cancer types. More interestingly, *FARS2* is down-regulated in KICH, while the two paralog ARSs, *FARSA* and *FARSB*, are up-regulated in six and four cancer types, respectively. The inconsistent between cy-ARSs and mt-ARSs may involve in metabolic pathway in tumor development. Further studies on ARSs, especially mt-ARSs are necessary to understand their roles in tumorigenesis. Taken together, the overexpression of ARSs may facilitate the accelerated charging process of tRNAs.

**Fig. 6:**
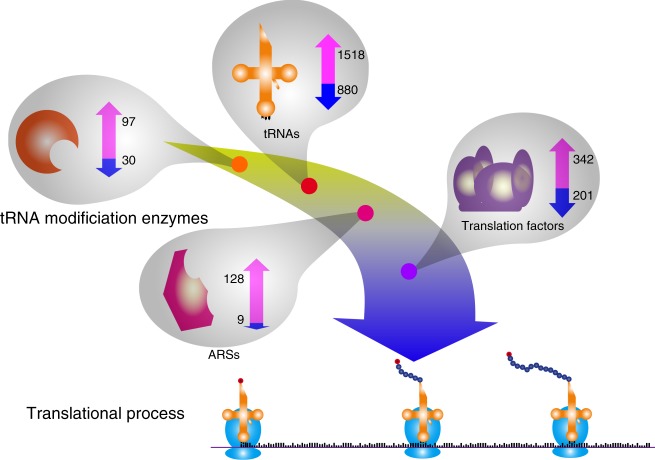
Synergistic activation of translation system in cancer. Overall overexpression of tRNAs, tRNA modification enzymes, ARSs, and translation factors across different cancer types, suggests the synergistic activation of translation system in cancer. Magenta and blue arrows denote number of up-regulated and down-regulated tRNA/enzyme, respectively

We further investigated the translation factors across cancer types and observed an overall overexpression and amplification pattern that 342 enzymes are up-regulated versus 201 down-regulated enzymes (two-sided *χ*^2^ test, *p* = 2.1 × 10^–5^, Fig. 5a, right panel). We observed up-regulation of several enzymes, including *EIF4EBP1* and *EIF3B*, and down-regulation, including *EIF4E3* and *EIF1AY* (Fig. 5d). Interestingly, *EEF1A2* has been reported to be overexpressed in multiple cancer types, including breast cancer and liver cancer^57,58^, while our analyses showed that *EEF1A2* is up-regulated in six cancer types, but strikingly down-regulated in four cancer types, namely COAD, esophageal carcinoma, head and neck squamous cell carcinoma, and stomach adenocarcinoma, suggesting a potential controversial function of *EEF1A2* in these cancer types. Consistent with the overall overexpression pattern, we also observed overall copy number amplifications for translation factors, including *EIF3H*, *EEF1D*, and *EIF3E* (Supplementary Figure 7). Taken together, the overall overexpression of translation factors may promote the translational process. In summary, we showed overall overexpression and copy number amplification for tRNA modification enzymes, ARSs, and translation factors across multiple cancer types, which may play synergistic roles with the overall overexpression of tRNAs (Fig. 6).

## Discussion

tRNAs can play important roles in cancer by accelerating the translational regulation and by supplying molecules that are in high demand for tumor metabolism^14,15^. Here, we developed a computational pipeline to infer the relative tRNA expression levels from miRNA-seq data from ~10,000 patient samples from TCGA across 31 cancer types. This is the first time to our knowledge that tRNAs have been analyzed in large-scale cancer samples, and provides a unique opportunity as the same samples have been comprehensively characterized by TCGA at the DNA, RNA, protein, pathological, and clinical levels. Our analysis achieved the highest resolution to date of tRNA expression to individual tRNAs, and showed overexpression of tRNAs across multiple cancer types. We further demonstrated the unequal alterations of tRNAs at the tRNA, codon, and amino acid levels. First, tRNA alterations in opposite directions may compensate for each other. Second, having too few differentially expressed tRNA genes may limit diversity of expression at the codon or amino acid level. These findings indicate that alterations in the expression of tRNA genes do not necessarily result in alterations at the codon and amino acid levels to influence translational regulation.

A previous study showed that tRNA overexpression in tumors may increase the translational efficiency of genes relevant to cancer development^18^. Interestingly, we observed that those codons that tend to be overexpressed in cancer samples exhibited significantly lower O/E ratios than those codons that tend to be down-regulated. Furthermore, the O/E ratios of amino acids tend to be negatively correlated with alterations in expression between tumor and normal samples. These observations suggest that overexpression of tRNAs from codons and amino acids with low O/E ratios may overcome a bottleneck posed to tumor development by the process of increased protein translation. Furthermore, overexpression of tRNA^Arg^ will promote breast cancer metastasis^17^, while we observed overexpression of tRNA^Arg^ in multiple cancer types. Our work laid the groundwork for an integrated functional interpretation to illuminate the functional roles of tRNAs.

Finally, we identified a series of enzymes with alterations across multiple cancer types, including unprecedented tRNA modification enzymes, such as those encoded by *PUS1*, *TRMT1*, and *TRMT6*. Particularly, we revealed the global gene overexpression of mt-ARSs, and individual mt-ARS exhibit divergent alterations with their cytosolic paralogs. More interestingly, we observed global overexpression and amplification of tRNAs, tRNA modification enzymes, ARSs, and translation factors. Thus, overexpression of tRNA modification enzymes stabilize tRNAs in order to increase the expression level of tRNAs. Overexpression of tRNAs and ARSs may accelerate the process of aminoacyl tRNA synthesis. Overexpression of translation factors may accelerate the translational initiation and translational elongation. Taken together, overexpression of tRNAs and enzymes involved in translational regulation highlights the synergistic activation of protein translation in cancer.

## Methods

### Quantitation of tRNA expression across different cancers

Supplementary Figure 1 shows a schematic of the overall analysis pipeline. The TCGA data portal was accessed to download miRNA-seq data 16,591 samples (https://portal.gdc.cancer.gov/) on June 2016. The latest version was kept if there were multiple BAM files for one sample. We calculated the number of quality control (QC)-passed reads and the number of reads mapped to human genome by samtools^59^. The lower-quality samples (i.e., those with fewer than 50% that passed QC or fewer than 80% of reads that mapped to the human genome) were filtered out. After filtering out the repeated bam files, we obtained 9931 patient tumor samples across 31 cancer types and their related 663 non-tumor tissue samples for use in the analyses. tRNA annotations (hg19) were downloaded from the UCSC Genome Browser (http://hgdownload.soe.ucsc.edu/). We calculated reads counts for each tRNA across all samples, and then quantified tRNA expression value using the TMM method^60,61^ following the steps from a previous study^62^.

In brief, we calculated the reads counts (*Y*_*gk*_) for each gene and the total number of reads (*N*_*k*_) for each sample. TMM introduced *M* value as

$$M_g = {\mathrm{log}}_2\left( {\frac{{\frac{{Y{gk}}}{{Nk}}}}{{\frac{{Y{gk}\prime }}{{Nk\prime }}}}} \right),$$

then was estimated as

log_2_(TMM) by $\frac{{\mathop {\sum }\nolimits_r^G w_{gk}^rM_{gk}^r}}{{\mathop {\sum }\nolimits_r^G w_{gk}^r}}.$ where $w_{gk}^r = \frac{{Nk - Y{gk}}}{{NkY{gk}}} + \frac{{N{r} - Y{gr}}}{{N{r}Y{gr}}}$.

TMM is implemented as the TMM module in the edgeR R Bioconductor. tRNAs with an average TMM ≥ 1 across samples in each cancer type were defined as detectable tRNAs (Supplementary Figure 1). We downloaded the tRNA expression data for DM-tRNA-seq in cell line from gene expression omnibus (GEO, GSE97259). We measured distance by Euclidean distance based on tRNA expression, and then classified tRNAs using unweighted pair-group method with arithmetic means method^63^.

### Analysis of tRNA expression at tRNA, codon, and amino acid levels

We used paired Student's *t* test to perform differentially expressed analyses in those cancer types with ≥ 5 paired tumor and normal samples, which normal samples are extracted from the adjacent tissues (Supplementary Table 1). For each cancer type, we estimated the *p* value of each tRNA between tumor and normal samples and then adjusted the *p* value by FDR (Benjamini–Hochberg procedure). We identified differentially expressed tRNAs with |fold-change| ≥ 1.5 and FDR < 0.05 considered to be significantly up-regulated or down-regulated. The detailed information for accepted amino acid accepted by each codon can be viewed at http://www.cbs.dtu.dk/courses/27619/codon.html. We then merged tRNA expression at codon and amino acid levels based on strict Watson-Crick match considering |fold-change| ≥ 1.5 and FDR < 0.05 to be significant. By downloading the original data, the interested reader can reproduce the calculations for other degrees of stringency. Cancer types with more up-regulated or down-regulated tRNAs, codons, and amino acids (fold-change ≥ 1.5) were considered as predominantly up-regulated or down-regulated cancer types, respectively. The O/E ratio was estimated by observed value/expected value (http://www.tiem.utk.edu/~gross/bioed/webmodules/aminoacid.htm). The observed value is the frequency of an amino acid or codon in the human genome. The expected frequency of a particular codon can be calculated by multiplying the frequencies of each DNA base comprising the codon. The expected frequency of the amino acid can then be calculated by adding the frequencies of each codon that codes for the amino acid.

Overall survival times for patient samples were obtained from TCGA’s data portal (https://tcga-data.nci.nih.gov/tcga). We used the univariate Cox model to test relationship between overall survival time and tRNA expression. We also used two-sided log-rank model to test the difference of survival time between two groups, for example, high tRNA expression group and low tRNA expression group. We considered FDR < 0.05 as statistically significant.

### Analysis of enzymes involved in translational regulation

tRNA modification enzymes were collected from the Modomics database (http://modomics.genesilico.pl/)^64^ and from the literature^6,31,65^. ARSs and translation factors were collected from previous studies^39,66–69^. Gene expression and CNV data were downloaded from TCGA’s data portal (https://tcga-data.nci.nih.gov/tcga/). Genes were considered to be differentially expressed if the |fold-change| ≥ 1.5 and FDR < 0.05. TCGA CNV scores for each gene were downloaded from a previous study^70^, CNV score > log_2_(3) or < log_2_(1) were defined as gain or loss, respectively^70^.

### Code availability

Custom scripts are available upon request.

## Supplementary information

Description of  Additional Supplementary Files

Supplementary Data

Supplementary Information

## Data Availability

All datasets of the current study are freely available in Synapse (https://www.synapse.org, syn8367000).
