# Supplementary material for: Global analysis of tRNA and translation factor expression reveals a dynamic landscape of translational regulation in human cancers
Source: Commun Biol. 2018 Dec 21;1:234. doi: 10.1038/s42003-018-0239-8 (PMC6303286; doi:10.1038/s42003-018-0239-8)
Supplement: Supplementary file 1 — Description of Additional Supplementary Files [file 42003_2018_239_MOESM1_ESM.docx]

**Description of Additional Supplementary Files**

**File Name**: Supplementary Data 1

**Description**: tRNA genes differentially expressed across different cancer types. Related to Figure 2.

**File Name**: Supplementary Data 2

**Description**: Differentially expressed tRNAs at the codon level. Related to Figure 3.

**File Name**: Supplementary Data 3

**Description**: Differentially expressed tRNAs at the amino acid level. Related to Figure 4.

**File Name**: Supplementary Data 4

**Description**: Related to Figure 5 and Figure 6.
