# Supplementary material for: Global analysis of tRNA and translation factor expression reveals a dynamic landscape of translational regulation in human cancers
Source: Commun Biol. 2018 Dec 21;1:234. doi: 10.1038/s42003-018-0239-8 (PMC6303286; doi:10.1038/s42003-018-0239-8)
Supplement: Supplementary file 3 — Supplementary Information [file 42003_2018_239_MOESM3_ESM.pdf]

Supplementary Figures

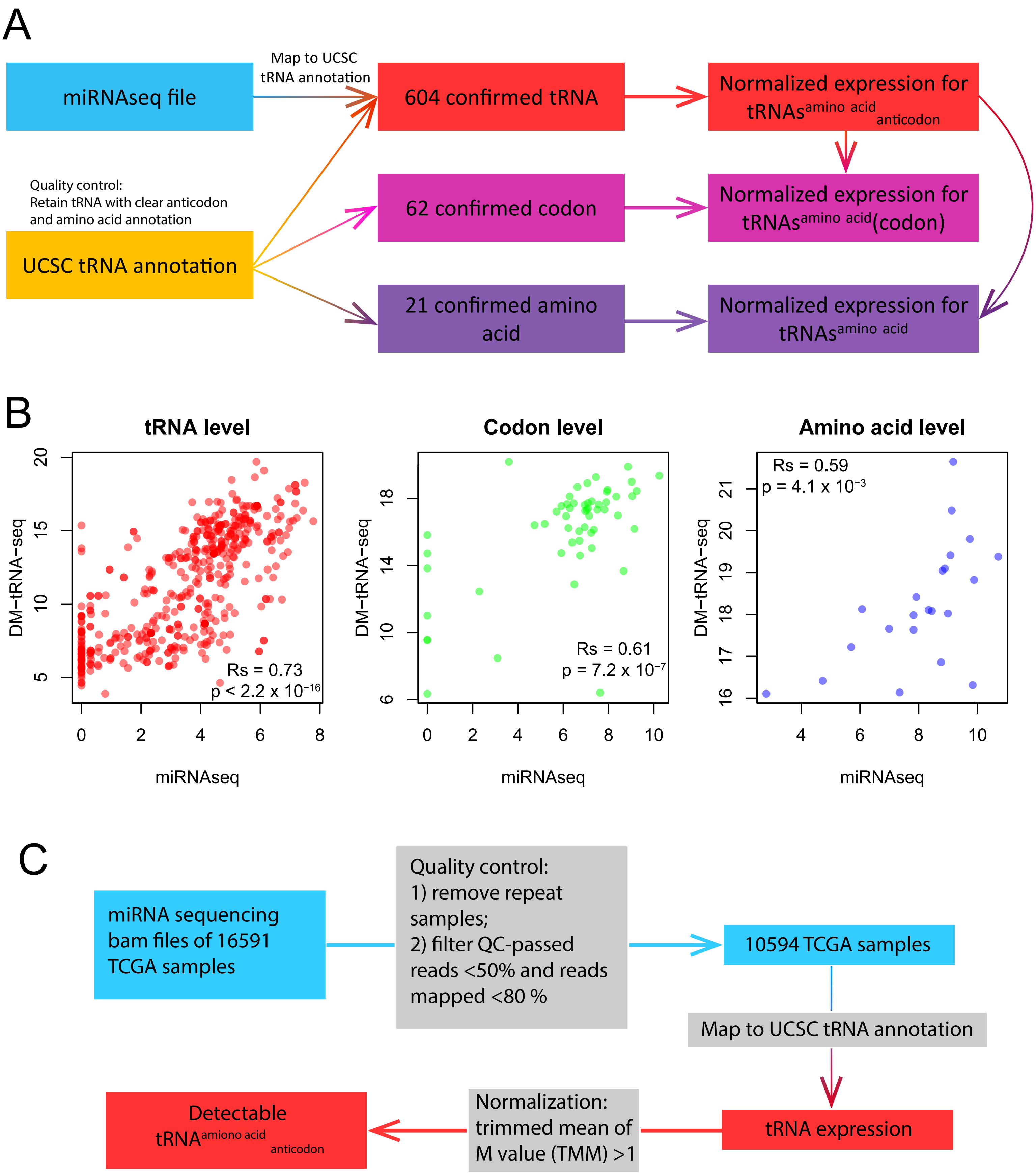

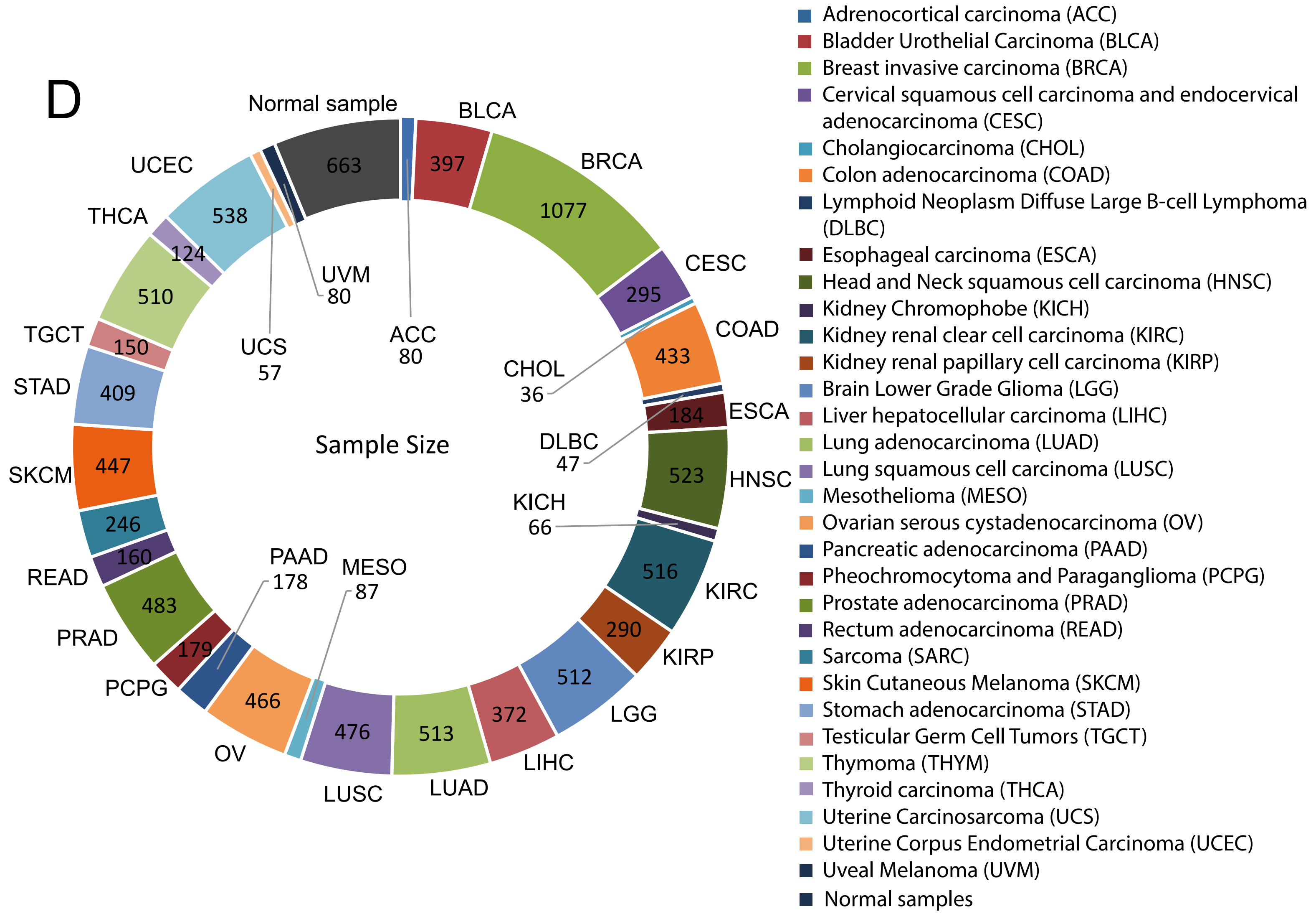

Supplementary Figure 1. Computational pipeline to quantify tRNA expression profiles.

(A) Computational pipeline to quantify tRNA expression profiles at gene, codon and amino acid levels.

(B) Correlation of tRNA expression between our computational pipeline based on miRNAseq and tRNA-DM-seq at tRNA level (left panel), codon level (middle panel) and amino acid level (right panel).

(C) Computational pipeline to process TCGA miRNAseq samples.

(D) Number of tumor and normal samples across different cancer types.

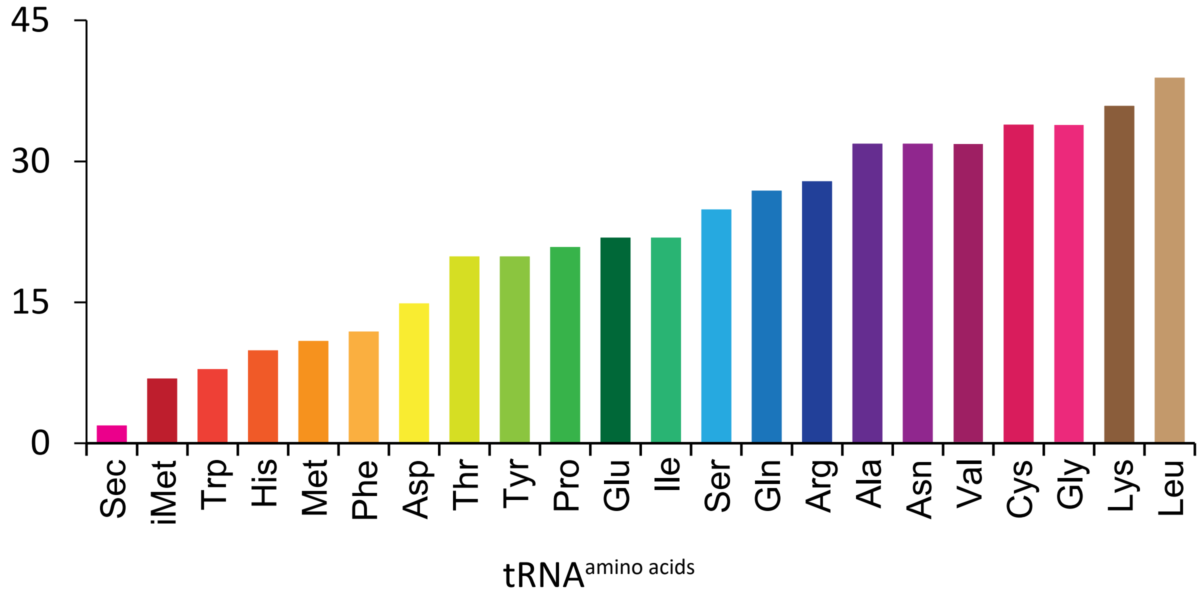

Supplementary Figure 2. Number of detectable tRNA genes for each amino acid.

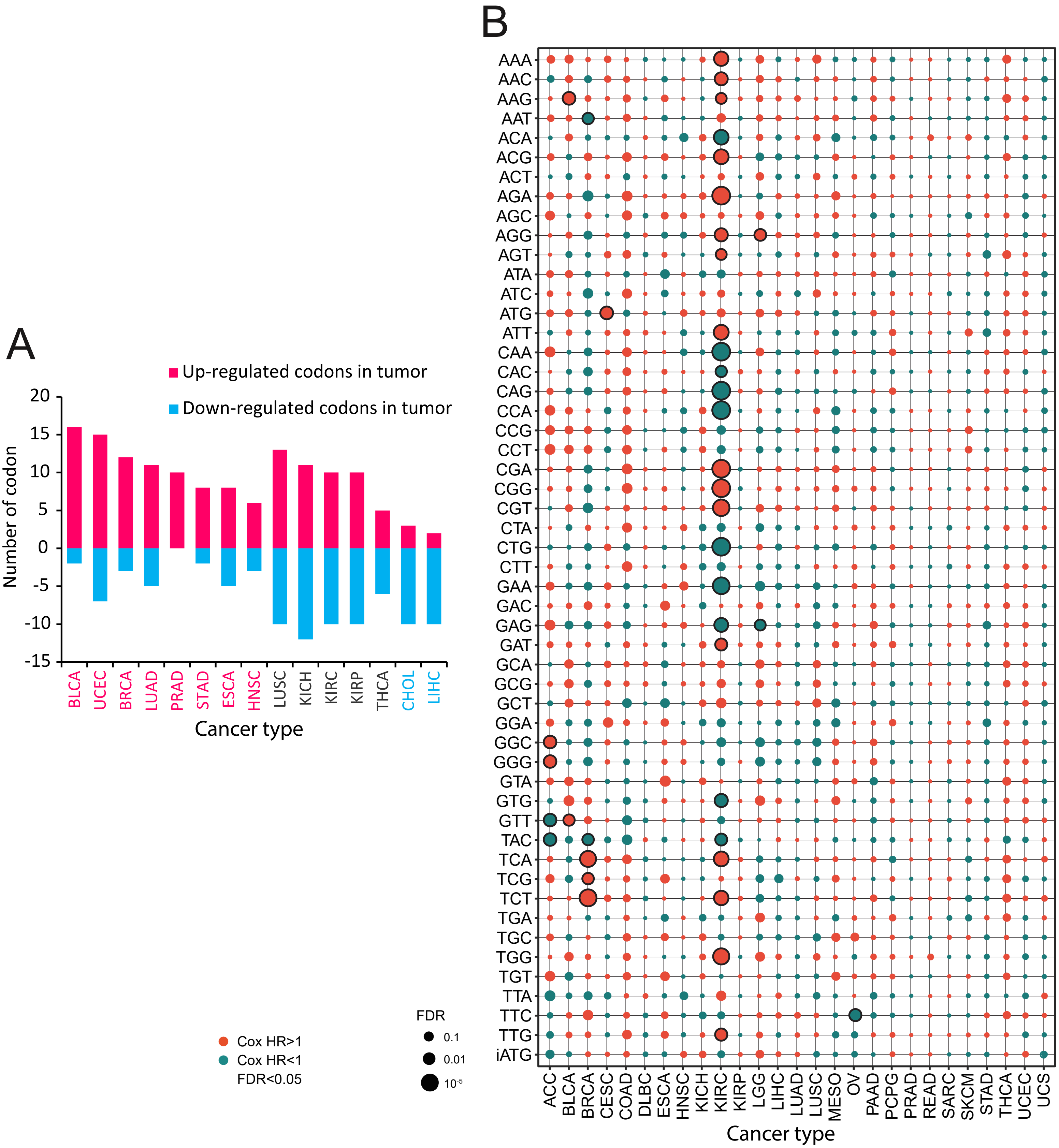

A
Up-regulated codons in tumor
Down-regulated codons in tumor
Number of codon
20
15
10
5
0
-5
-10
-15
BLCA
UCEC
BRCA
LUAD
PRAD
STAD
ESCA
HNSC
LUSC
KICH
KIRC
KIRP
THCA
CHOL
LIHC
Cancer type
B
AAA
AAC
AAG
AAT
ACA
ACG
ACT
AGA
AGC
AGG
AGT
ATA
ATC
ATG
ATT
CAA
CAC
CAG
CCA
CCG
CCT
CGA
CGG
CGT
CTA
CTG
CTT
GAA
GAC
GAG
GAT
GCA
GCG
GCT
GGA
GGC
GGG
GTA
GTG
GTT
TAC
TCA
TCG
TCT
TGA
TGC
TGG
TGT
TTA
TTC
TTG
iATG
ACC
BLCA
BRCA
CESC
COAD
DLBC
ESCA
HNSC
KICH
KIRC
KIRP
LGG
LIHC
LUAD
LUSC
MESO
OV
PAAD
PCPG
PRAD
READ
SARC
SKCM
STAD
THCA
UCEC
UCS
Cancer type
Cox HR>1
Cox HR<1
FDR<0.05
FDR
0.1
0.01
10-5

Supplementary Figure 3. Differentially expressed Codons across cancer types and functional analyses.

(A) Number of up-regulated and down-regulated codons across different cancer types. X-axis represents 15 cancer types with > 5 tumor-normal paired samples.

(B) Survival analyses based on univariate Cox model across different cancer types. Size of circle denotes FDR; circle color denotes hazard ratio (HR). The border denotes adjusted $p < 0.05$.

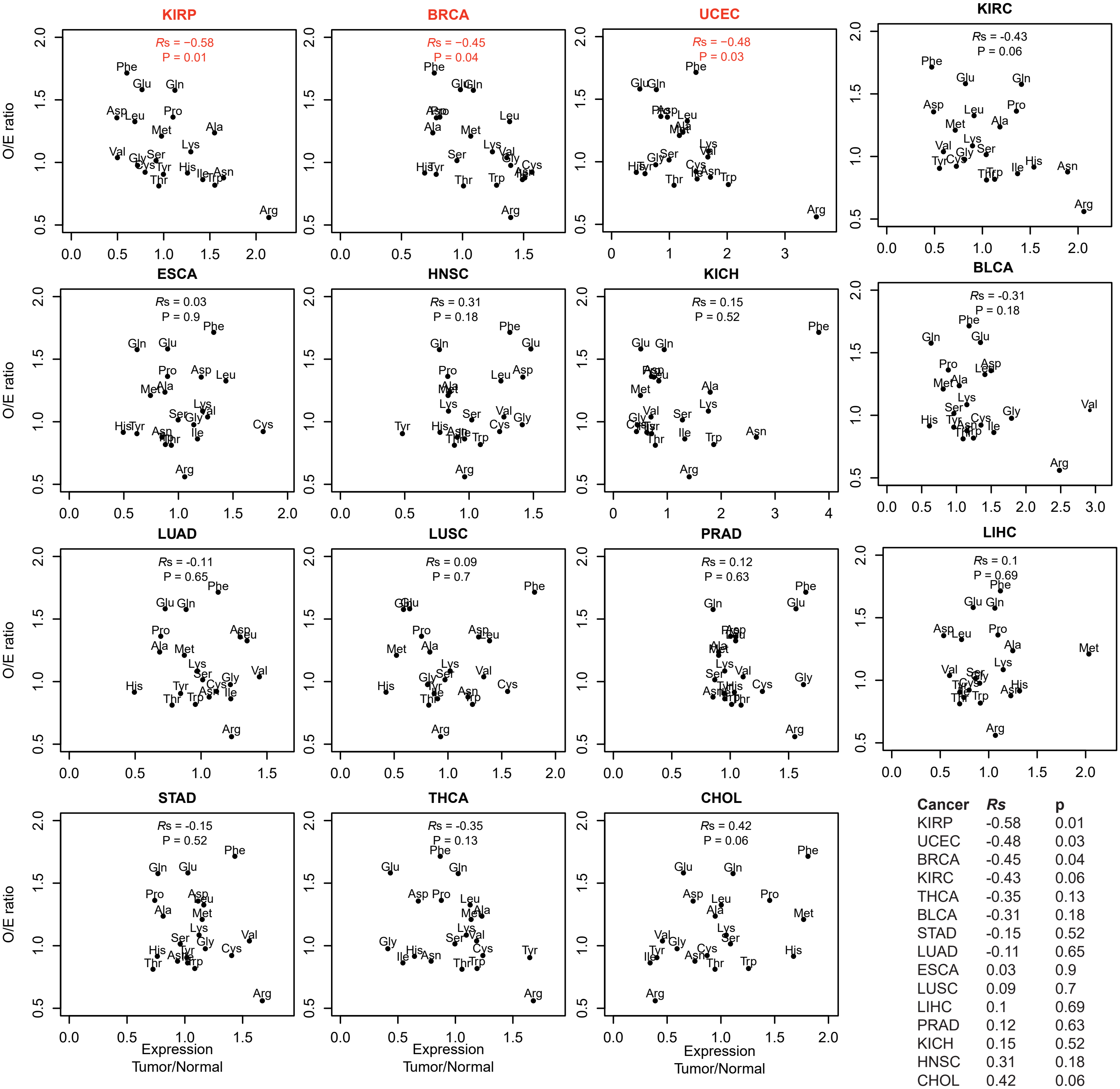

Cancer	*Rs*	p
KIRP	-0.58	0.01
UCEC	-0.48	0.03
BRCA	-0.45	0.04
KIRC	-0.43	0.06
THCA	-0.35	0.13
BLCA	-0.31	0.18
STAD	-0.15	0.52
LUAD	-0.11	0.65
ESCA	0.03	0.9
LUSC	0.09	0.7
LIHC	0.1	0.69
PRAD	0.12	0.63
KICH	0.15	0.52
HNSC	0.31	0.18
CHOL	0.42	0.06

Supplementary Figure 4. Spearman correlation between amino acid O/E ratio and tRNA expression alteration.

Spearman correlation between amino acid O/E ratio (Y-axis) and tRNA expression alteration (X-axis) at amino acid level in 15 cancer types. Red color denotes the significant correlation.

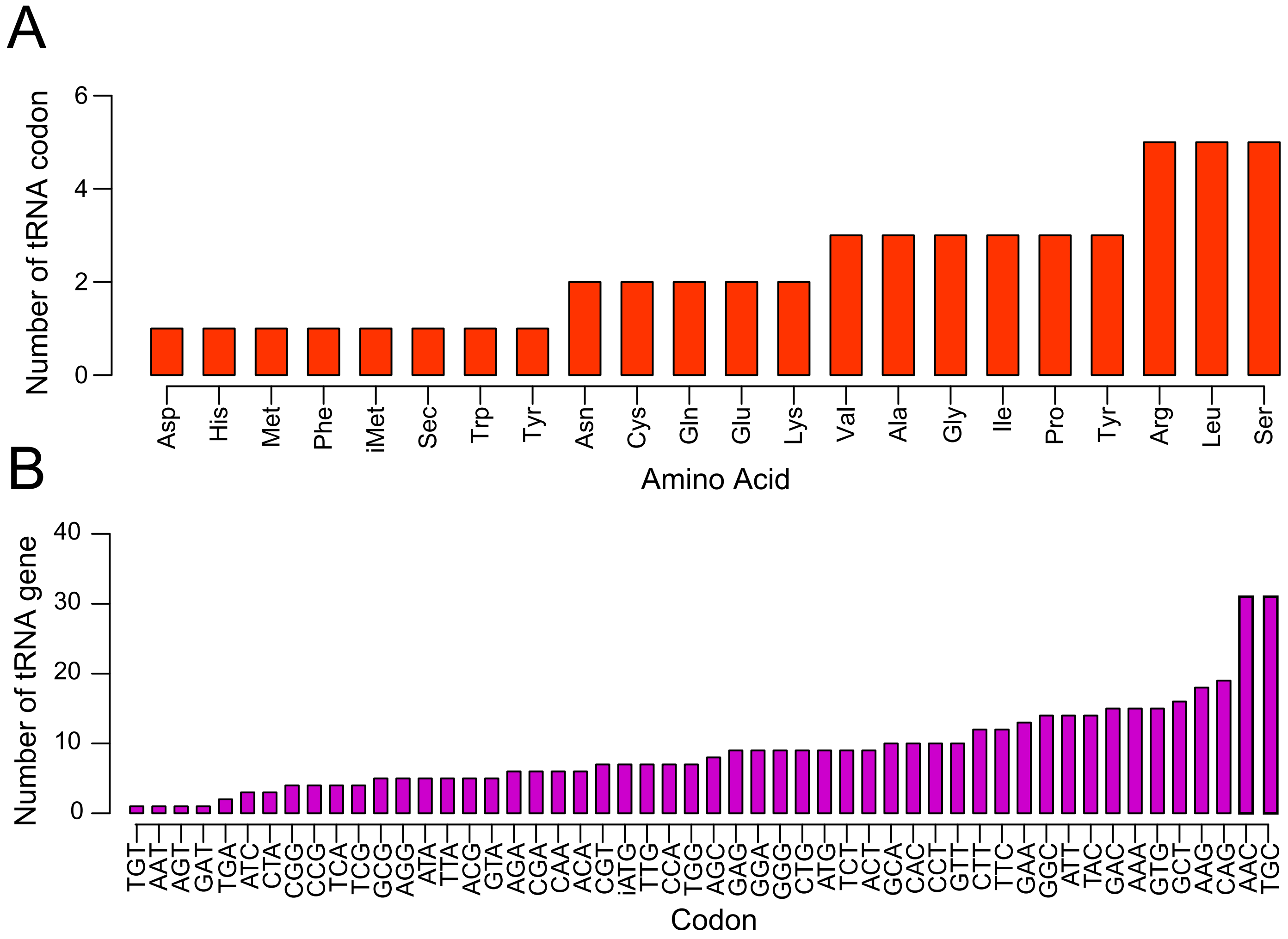

Supplementary Figure 5. Information for detectable tRNAs

(A) Number of detectable tRNA codons for each amino acid.

(B) Number of detectable tRNA genes for each codon.

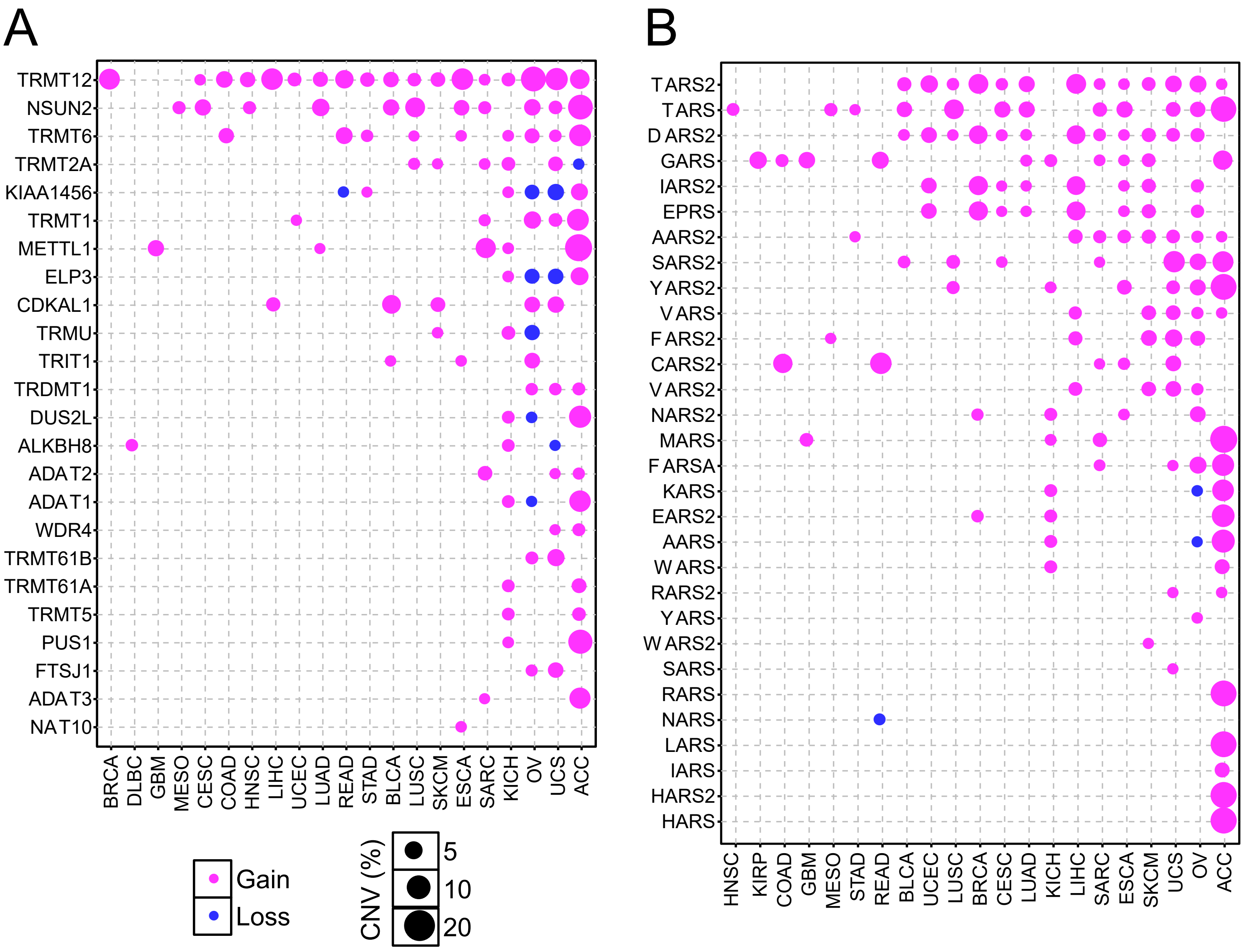

Supplementary Figure 6. CNV analyses for tRNA-related enzymes across different cancer types.

(A and B) Copy number variation gains (magenta) and losses (blue) of tRNA modification enzymes (A) and ARSs (B), respectively. Circle size indicates the percentage of samples with altered CNVs. X-axis represents cancer types with at least one enzyme showed copy number variations in at least 5% samples.

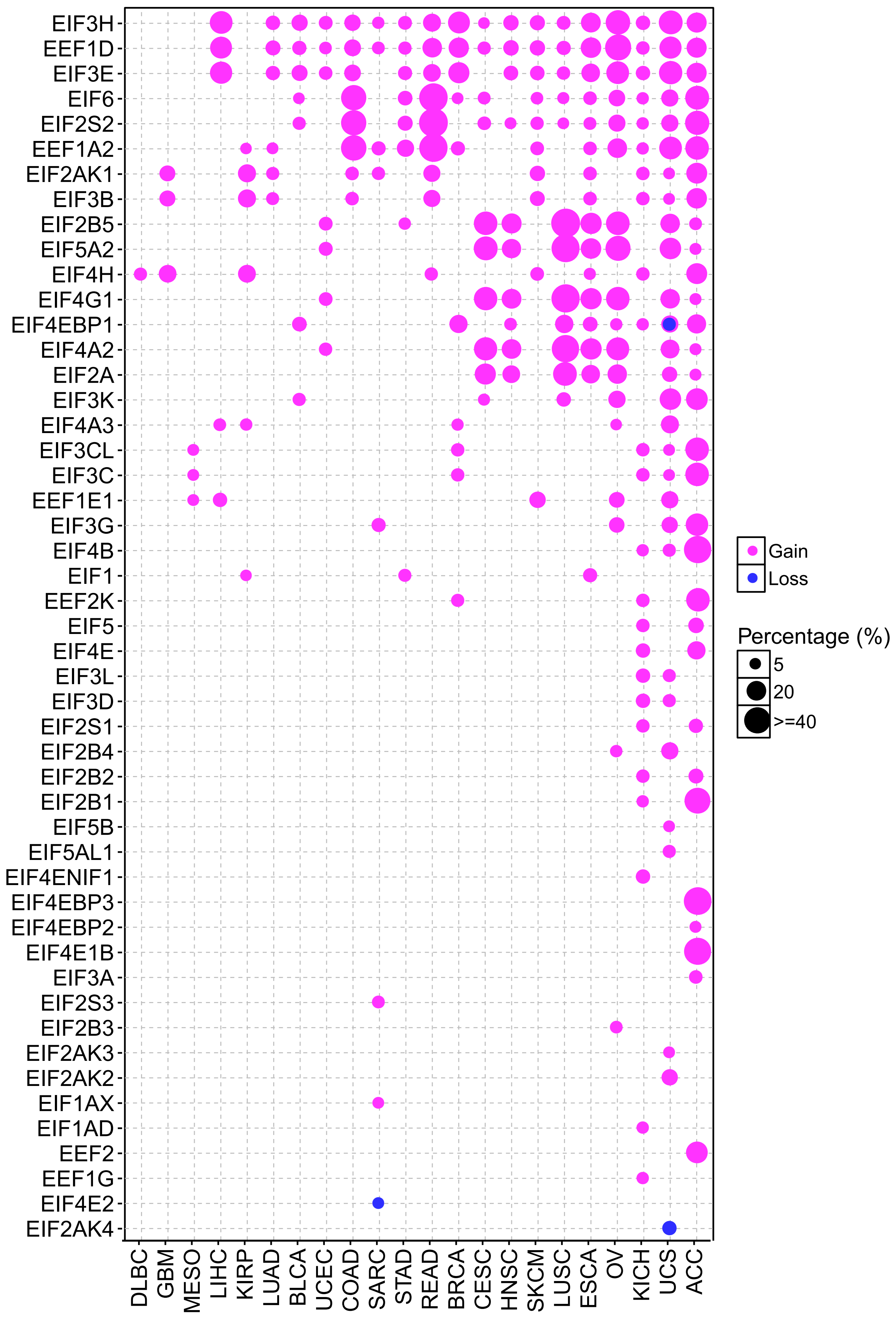
EIF3H
EEF1D
EIF3E
EIF6
EIF2S2
EEF1A2
EIF2AK1
EIF3B
EIF2B5
EIF5A2
EIF4H
EIF4G1
EIF4EBP1
EIF4A2
EIF2A
EIF3K
EIF4A3
EIF3CL
EIF3C
EEF1E1
EIF3G
EIF4B
EIF1
EEF2K
EIF5
EIF4E
EIF3L
EIF3D
EIF2S1
EIF2B4
EIF2B2
EIF2B1
EIF5B
EIF5AL1
EIF4ENIF1
EIF4EBP3
EIF4EBP2
EIF4E1B
EIF3A
EIF2S3
EIF2B3
EIF2AK3
EIF2AK2
EIF1AX
EIF1AD
EEF2
EEF1G
EIF4E2
EIF2AK4
DLBC
GBM
MESO
LIHC
KIRP
LUAD
BLCA
UCEC
COAD
SARC
STAD
READ
BRCA
CESC
HNSC
SKCM
LUSC
ESCA
OV
KICH
UCS
ACC
Gain
Loss
Percentage (%)
5
20
>=40

Supplementary Figure 7. CNV analyses for translational factors across different cancer types.

Copy number variation gains (magenta) and losses (blue) of translation factors. Circle size indicates the percentage of samples with altered CNVs. X-axis represents cancer types with at least one enzyme showed copy number variations in at least 5% samples.

Supplementary Tables

Supplementary Table 1. Number of samples and detailed abbreviation name for each cancer type.

Cancer type	Abbrevia☒on name	# of tumor samples (miRNA)	# of normal samples (miRNA)	# of paired samples (miRNA)	# of tumor samples (mRNA)	# of normal samples (mRNA)	# of paired samples (mRNA)
Adrenocortical carcinoma	ACC	80	0	0	79	0	0
Bladder Urothelial Carcinoma	BLCA	397	16	16	408	19	19
Breast invasive carcinoma	BRCA	1077	104	104	1084	99	99
Cervical squamous cell carcinoma and endocervical adenocarcinoma	CESC	295	3	3	298	3	3
Cholangiocarcinoma	CHOL	36	9	9	36	4	4
Colon adenocarcinoma	COAD	433	1	1	463	41	41
Lymphoid Neoplasm Diffuse Large B-cell Lymphoma	DLBC	47	0	0	47	0	0
Esophageal carcinoma	ESCA	184	11	11	184	11	11
Head and Neck squamous cell carcinoma	HNSC	523	44	44	497	44	44
Kidney Chromophobe	KICH	66	25	25	65	24	24
Kidney renal clear cell carcinoma	KIRC	516	71	71	530	72	72
Kidney renal papillary cell carcinoma	KIRP	290	34	34	287	32	32
Brain Lower Grade Glioma	LGG	512	0	0	501	0	0
Liver hepatocellular carcinoma	LIHC	372	50	50	369	51	51
Lung adenocarcinoma	LUAD	513	46	46	517	57	57
Lung squamous cell carcinoma	LUSC	476	45	45	497	49	49
Mesothelioma	MESO	87	0	0	81	0	0
Ovarian serous cystadenocarcinoma	OV	466	0	0	354	0	0
Pancreatic adenocarcinoma	PAAD	178	4	4	178	4	4
Pheochromocytoma and Paraganglioma	PCPG	179	3	3	177	3	3
Prostate adenocarcinoma	PRAD	483	52	52	483	51	51
Rectum adenocarcinoma	READ	160	0	0	163	10	10
Sarcoma	SARC	246	0	0	259	2	2
Skin Cutaneous Melanoma	SKCM	447	2	2	468	1	1
Stomach adenocarcinoma	STAD	409	37	37	373	32	32
Testicular Germ Cell Tumors	TGCT	150	0	0	149	0	0
Thyroid carcinoma	THCA	510	71	71	504	56	56
Thymoma	THYM	124	2	2	119	2	2
Uterine Corpus Endometrial Carcinoma	UCEC	538	33	33	541	35	35
Uterine Carcinosarcoma	UCS	57	0	0	56	0	0
Uveal Melanoma	UVM	80	0	0	77	0	0
